# The p50 Subunit of NF-κB Orchestrates Dendritic Cell Lifespan and Activation of Adaptive Immunity

**DOI:** 10.1371/journal.pone.0045279

**Published:** 2012-09-25

**Authors:** Paola Larghi, Chiara Porta, Elena Riboldi, Maria Grazia Totaro, Lorenzo Carraro, Ciriana Orabona, Antonio Sica

**Affiliations:** 1 Humanitas Clinical and Research Center, Rozzano, Milan, Italy; 2 Dipartimento di Scienze del Farmaco, Università del Piemonte Orientale “A. Avogadro”, Novara, Italy; 3 Department of Experimental Medicine and Biochemical Sciences, Università di Perugia, Perugia, Italy; University of Torino, Italy

## Abstract

Dendritic cells play a central role in keeping the balance between immunity and immune tolerance. A key factor in this equilibrium is the lifespan of DC, as its reduction restrains antigen availability leading to termination of immune responses. Here we show that lipopolysaccharide-driven DC maturation is paralleled by increased nuclear levels of p50 NF-κB, an event associated with DC apoptosis. Lack of p50 in murine DC promoted increased lifespan, enhanced level of maturation associated with increased expression of the proinflammatory cytokines IL-1, IL-18 and IFN-β, enhanced capacity of activating and expanding CD4^+^ and CD8^+^ T cells *in vivo* and decreased ability to induce differentiation of FoxP3^+^ regulatory T cells. In agreement, vaccination of melanoma-bearing mice with antigen-pulsed LPS-treated p50^−/−^ BM-DC boosted antitumor immunity and inhibition of tumor growth. We propose that nuclear accumulation of the p50 NF-κB subunit in DC, as occurring during lipopolysaccharide-driven maturation, is a homeostatic mechanism tuning the balance between uncontrolled activation of adaptive immunity and immune tolerance.

## Introduction

Dendritic cells (DC) are professional antigen-presenting cells (APC) with remarkable functional plasticity that play an essential role in the balance between immunity and immune tolerance [1]–[3]. DC originate from the bone marrow and, at an “immature” stage, patrol peripheral tissues for the presence of pathogen-associated antigens to activate specific immunity [1]–[3]. In order to accomplish this program, DC express a rich repertoire of pattern recognition receptors, including Toll-like receptors (TLR), which allow DC to recognize distinct pathogen-associated molecules and to undergo a process of maturation [4], [5]. Maturation of DC is characterized by increased expression of the co-stimulatory molecules CD80 and CD86, synthesis of pro-inflammatory cytokines, higher CCR7-mediated migration into the T cell-rich regions of the lymph node and increased antigen-specific T cell activation [1].

Because of their unique role in linking the innate and adaptive immunity, DC-based immunotherapy is widely considered in clinical vaccination trials with cancer patients, predominantly with *ex vivo*-cultured monocyte-derived DC (mo-DC) [6]. However, major problems remain as the tumour microenvironment may express high levels of immunosuppressive cytokines (eg. IL-10, TGFβ) leading to an incomplete form of DC maturation with tolerogenic properties [7], [8]. As a consequence, a major challenge in optimizing DC-based immunotherapy is the identification of new mechanisms controlling DC maturation and their antigen presentation capacity, compatible with protective antitumor immune responses [9].

DC are key cells in maintaining intrathymic and peripheral tolerance [7] and in animal models their depletion is associated with the onset of fatal autoimmune-type diseases [10], [11]. Both myeloid and plasmacytoid immature DC have been described as inducers of T cell tolerance [2], [7], capable of inducing IL-10-producing regulatory T cells (Treg), anergy of T cells or activation-induced cell death [3]. Among the immunosuppressive mechanisms exploited by tolerogenic DC, expression of indoleamine 2,3-dioxygenase (IDO) appears to be the most powerful. IDO-mediated immune regulation occurs via both tryptophan starvation and production of the immunoregulatory catabolites kynurenines [12], which in turn promote differentiation of Foxp3^+^ regulatory T cells [13]. Additional evidence indicate that modulation of DC lifespan may efficiently control activation and extinction of the immune response. While reduction of DC lifespan restrains antigen availability for T cells, leading to termination of immune responses, prolonged DC survival results in perpetuation of adaptive immune reactions [14], [15].

NF-κB is a master regulator of inflammation and has been reported to play a key role in guiding DC maturation and immune functions [16], [17] and to mediate protection of DC from death caused by cytokines withdrawal [18]. However, no detailed analysis is available on the role played by different NF-κB subunits in DC survival. Unlike c-Rel, RelB, and RelA proteins, p50 and p52 family members do not contain the COOH-terminal transactivation domain and may form inhibitory homodimers that function as transcriptional repressors [19], [20]. Importantly, altered activation of selected NF-κB members has been reported in various pathologic conditions, including infection and cancer [19], [21]–[23]. In particular, we have shown that nuclear accumulation of p50 NF-κB promotes both tolerance in tumor-associated macrophages (TAM) [22] and M2 (alternative) macrophage polarization [19]. In agreement with these findings, we report now that p50 NF-κB promotes a tolerogenic phenotype in DC, affecting both their survival and capacity to drive effective activation of effector T cells.

## Materials and Methods

### Ethics Statement

The study was designed in compliance with principles set out in the following laws, regulations and policies governing the care and use of laboratory animals: Italian Governing Law (Legislative Decree 116 of Jan. 27, 1992); EU directives and guidelines (EEC Council Directive 86/609, OJ L 358, 12/12/1986); Legislative Decree September 19, 1994, n. 626 (89/391/CEE, 89/654/CEE, 89/655/CEE, 89/656/CEE, 90/269/CEE, 90/270/CEE, 90/394/CEE, 90/679/CEE); the NIH Guide for the Care and Use of Laboratory Animals (1996 edition); Authorization n. 11/2006-A issued January 23, 2006 by Ministry of Health. The study was approved by the scientific board of Humanitas Clinical and Research Center. Humanitas Clinical and Research Center Institutional Regulations and Policies providing internal authorization for persons conducting animal experiments. Mice have been monitored daily and euthanized when displaying excessive discomfort.

### Mice

p50 NF-κB-deficient mice were donated by Prof. Michael Karin [22]. Littermates were used as controls whereas OT-II mice were from Jackson Laboratories (Bar Harbor, Maine, USA). The study was designed in compliance with the National Institutes of Health Guidelines for the Care and Use of Laboratory Animals and European Union directives and guidelines.

### Cell Culture and Reagents

DC were derived from bone marrows (BM-DC) of p50^−/−^ or wt C57/BL6J mice cultured in IMDM containing 10% FBS, supplemented with 30% supernatant from granulocyte macrophage colony-stimulating factor–producing NIH-3T3 cells [24]. BM-DC were either left untreated (immature), or treated with LPS as a stimulus to induce a mature phenotype. B16-OVA cells were kindly provided by Prof. P. Dellabona (San Raffaele Scientific Institute, Milan). Highly enriched DC (>90%) were obtained from spleens by selection with CD11c-conjugated microbeads (Miltenyi Biotec, Germany). LPS (100 ng/ml; LPS from *Salmonella Abortus Equi* S-form; Alexis), IFN-γ (200 U/ml; Peprotech), ovalbumin (OVA; 80 µg/ml; Sigma Aldrich), OVA_323–339_ and OVA_257–264_ peptides (0.1 µg/mL; ANASPEC, Fremont, CA). DQ-ovalbumin (0,2 mg/ml; Molecular Probes).

#### Western blot analysis

Proteins were analyzed by SDS-PAGE as previously described [22]. Antibodies: anti-p50 antibody [7], anti-Bax (Cell Signaling Technology, Danvers, MA), anti-actin (Sigma Aldrich).

### FACS Analysis

Cells were stained with: anti-CD11c, anti-CD45 from Biolegend, San Diego, CA; anti-I-A/I-E, anti-H2-K^b^, anti-CD8a, anti-CD4, anti-CD25, anti-IFN-γ from BD Biosciences, San Diego, CA; anti-Foxp3 (e-Bioscience, San Diego, CA). For dexamer staining of OVA-specific CD8^+^ T cells: APC conjugated anti-mouse antibody against H-2K^b^ (SIINFEKL) (DAKO, Glostrup, Denmark). For PI/AnnexinV staining, the AnnexinV-FITC apoptosis detection kit (Immunostep, Salamanca, Spain) was used.

### Enzyme-linked Immunosorbent Assay (ELISA)

Murine IL-12p70, IFN-γ, IL-1β ELISA kits were purchased from R&D Systems (Minneapolis, MN). IL-18 and IFN-β ELISA kits were from MBL International Corporation (Woburn, MA).

### Macropinocytosis

BM-DC, 10^6^/ml in IMDM medium, were incubated with dextran-FITC (2 mg/ml; Sigma Aldrich) at 37°C. As negative controls for micropinocytosis, cells were incubated in ice; To determine cells autofluorescence, cells were also incubated at 37°C without dextran-FITC. Cells were than extensively washed and FITC fluorescence was analyzed by FACS.

### Antigen Processing Assay

BM-DC were harvested and resuspended in medium with 5% FCS at the concentration of 10^7^/mL. DQ-OVA (FITC) was added to the cells and incubated for 1 h at 37°C. Cells were then extensively washed and resuspended in medium at the concentration of 10^6^/mL. 100µL of cells were seeded in triplicates in 96 well plates at 37°C. Control 96 well plate was incubated in ice. At the indicated time points, cells were transferred in ice to block protein processing and FITC fluorescences was analyzed by FACS. Antigen processing was expressed as fold increase of M.F.I. compared to control cells at time zero.

### *In vitro* Secretion of IFN-γ by T Cells

BM-DC were left untreated or LPS was added to BM-DC cultures for 24 hours. OVA_323–339_ was added for the last 2 hours of culture. CD4^+^ T cells were purified from spleens of OT-II mice by positive selection with CD4-conjugated microbeads (Miltenyi Biotec). BM-DC were washed and resuspended in medium. Co-cultures of 3×10^4^ BM-DC and 10^5 ^T cells were seeded in 96 multiwell plates for 5 days. Supernatants were then tested for IFN-γ production.

### *Ex vivo* Secretion of IFN-γ by T Cells

BM-DC were left untreated or LPS was added to BM-DC cultures for 24 hours; OVA_257–264_ or OVA_323–339_ was added to the cultures for the last 2 hours. BM-DC were harvested and resuspended in PBS and 10^6^ BM-DC were injected subcutaneously in the hind leg footpad of wild type mice. Popliteal lymph nodes were recovered 7 days later, mechanically processed and resuspended at the concentration of 10^6^/mL in medium. 1 mL of the total suspension was then seeded in 24-well plates in the presence of OVA_257–264_ or OVA_323–339_ peptide. After 20 hours supernatants were collected and tested for the presence of IFN-γby ELISA. Cells were restimulated with 50 ng/ml PMA (Sigma), 1****µg/ml Ionomicin (Sigma) and Brefeldin A (e-Bioscience) for 4 h before intracell FACS analysis.

### *In vitro* BM-DC Migration

BM-DC migration was evaluated using a chemotaxis microchamber technique [25]. Briefly, 30µL of chemoattractant solution or control medium (RPMI 1640 with 1% FBS) was added to the lower wells of a chemotaxis chamber (Neuroprobe, Gaithersburg, MD) and a polycarbonate filter (5 µm pore size; Neuroprobe) was placed into the wells and covered with a silicon gasket. Cells were activated with LPS for 24 h and resuspended at 10^6^/mL. 50µL of cell suspension was seeded in the upper wells, and the chamber was incubated at 37°C for 90 minutes. At the end of this period, filters were removed and stained with Diff-Quik (Baxter, McGaw Park, IL).

### *In vivo* BM-DC Migration

LPS was added to BM-DC cultures for 24 h, cells were then harvested and labelled with 0.5 mM of CellTracker™ Orange CMTMR (5-(and-6)-(((4-chloromethyl)benzoyl)amino)tetramethylrhodamine)-mixed isomers (Molecular Probes, Life Technologies) for 10 minutes at 37°C. Cells were then extensively washed and resuspended in PBS. 10^6 ^BM-DC were injected subcutaneously in the hind leg footpad and popliteal lymph nodes were recovered 24 hours and 48 h later, mechanically disaggregated and treated with collagenase A (1 mg/mL; Boehringer Mannheim, Indianapolis, IN) and DNase (0.4 mg/mL; Roche, Indianapolis, IN) for 30 minutes; the enzymatically treated cell suspension was evaluated by FACS.

### *In vivo* DC Survival

Wt and p50^−/−^ mice showing an average of 70 million total spleen cells were injected intravenously with 100 ng/g (animal weight) of LPS or PBS. After 24 or 48 hours spleens were collected and total spleen cells stained for CD11c for subsequent FACS analysis. Splenic DC mortality was evaluated by fold increase of % of CD11c positive cells compared to PBS treated controls. *In situ* apoptosis of splenic DC was determined by TUNEL staining as previously described [26]. Slides were labeled as per TUNEL assay manufacturer’s instructions: QIA33 FragEL™ DNA Fragmentation Detection Kit, Colorimetric - TdT Enzyme (Calbiochem, EMD4Biosciences, Gibbstown, NJ).

### Vaccination

BM-DC were incubated with ovalbumin (OVA) or irrelevant protein (BSA) for 6 hours and LPS was added to the culture for additional 18 hours. 5×10^5^ BM-DC were then injected intraperitoneally in wt recipient mice. 5×10^4^ B16-OVA melanoma cells were injected subcutaneously in the flank of the animal 12 days later and tumor growth was measured with a caliper starting from day 13 on. At day 30, spleens were collected, mechanically processed and resuspended in medium. 10^6^ cells of the total suspension were then seeded in 24-well plates for 72 hours in the presence of OVA_257–264_ peptide. Next, supernatants were collected and tested for the presence of IFN-γ by ELISA.

### *In vitro* Generation of Foxp3+ Cells

T cells were purified by immunomagnetic positive selection with CD4-conjugated microbeads (Miltenyi Biotec Inc. Auburn, CA) from spleen and lymph nodes of OT-II transgenic mice and were tested for naïve/memory phenotype (expression of CD62L and CD44 surface markers). 3×10^4^ BM-DC and 10^5^ naïve T cells were seeded in co-culture in 96 multiwell plates for 5 days in antigen unspecific manner or in the presence of OVA_323–339_ peptide. Percentage of Foxp3^+^ cells was evaluated by FACS.

### Kynurenine Production

IDO functional activity was measured in vitro in terms of splenic DC ability to metabolize tryptophan to kynurenine, the concentrations of which were measured by high-performance liquid chromatography (HPLC), as previously described [27].

### Silencing of p50 in Human DC

Highly enriched blood monocytes were obtained from buffy coats by Ficoll and Percoll gradients (GE Healthcare, Uppsala, Sweden) as previously reported [28]. Monocytes were cultured at 1 x 10^6^/ml in RPMI 1640 w/o antibiotics complemented with 2 mM glutammine, 10% FCS, 50 ng/ml GM-CSF, and 20 ng/ml IL-4. After 48 h, differentiating cells were transfected with a validated p50-specific Stealth RNAi siRNA (final concentration 200 nM, Invitrogen) or with a control Stealth RNAi siRNA (scrambled sequence, 200 nM, Invitrogen) using Opti-MEM I Reduced Serum Medium and Lipofectamine 2000 transfection reagent (Invitrogen) according to the manufacturer’s protocol. Transfected cells were incubated for 72 h, then matured with LPS (100 mg/ml) and further incubated for 48 h. Co-cultures of 2×10^4^ human DC and 2×10^5^ allogeneic T cells were seeded in 96 multiwell plates for 5 days. Supernatants were then tested for IFN-γ production by a specific ELISA kit (R&D Systems).

### Statistics

Data are presented as means ± standard error of mean (SEM). Statistical comparison between groups was determined by Student’s t test. *P<0.05; **P<0.01.

## Results

### p50 NF-κB Regulates BM-DC Antigen-presenting Capacity, *in vitro* and *in vivo*

**Figure 1:**
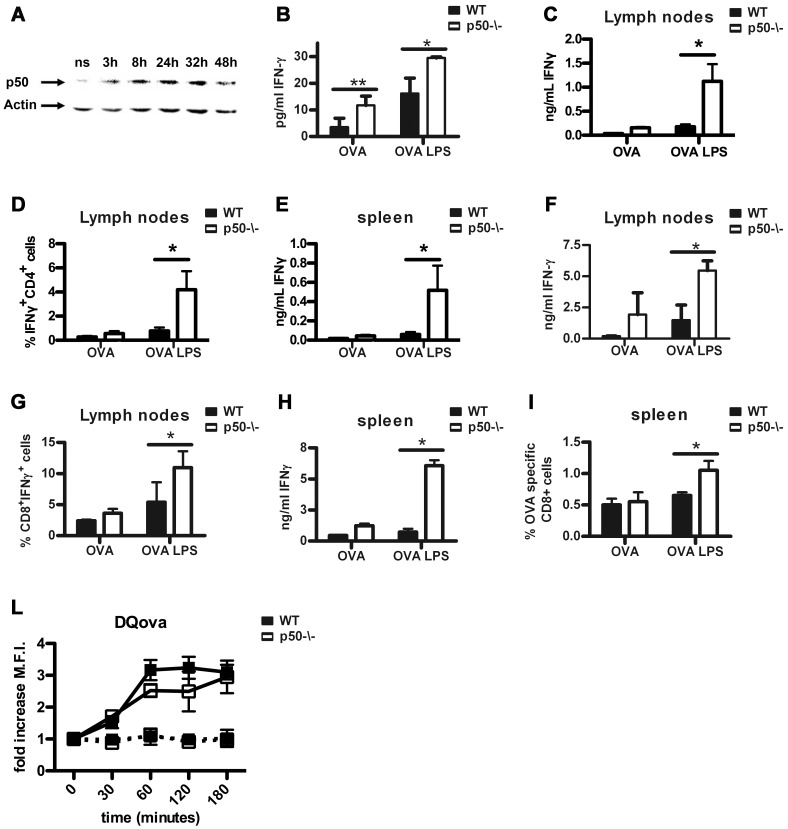
p50 NF-κB regulates the antigen-presenting capacity of DC. (A) Kinetic of nuclear accumulation of p50 NF-κB in wt BM-DC stimulated with LPS. One of 3 independent experiments with similar results is shown. (B) IFN-γ secretion by CD4+ T cell co-cultured with wt or p50^−/−^ untreated or LPS-treated BM-DC loaded with a MHC II-restricted peptide. Data represent mean ± SEM (N = 5). (C-E) wt or p50^−/−^ untreated or LPS-treated BM-DC loaded with a MHC II-restricted peptide were injected in the footpad of wt recipient mice. Next, *ex* vivo IFN-γ secretion by CD4+T cells from draining lymph nodes (C) and spleen (E) was measured. (F-I) wt or p50^−/−^ untreated or LPS-treated BM-DC loaded with a MHC I-restricted peptide were injected in the footpad of wt recipient mice. Next, *ex* vivo IFN-γ secretion by CD8+T cells from draining lymph nodes (F) and spleen (H) was measured. Lymph nodes and spleen were also analyzed for the presence of double positive IFNγ and CD4 (D) or CD8 cells (G) or OVA-specific CD8+ T cells (I). (L) wt or p50^−/−^ BM-DC were incubated with DQ ovalbumin at 37°C or 0°C and antigen processing was analyzed by mean of fold increase of M.F.I. compared to cells at time zero (dotted line: 0°C, straight line: 37°C). Data represent mean ± SEM (N = 2; 6 mice/group). * *P*<0.05 ** *P*<0.01, *t* test.

As increased nuclear levels of the p50 NF-κB subunit have been associated with lipopolysaccharide (LPS)-mediated tolerance of macrophages [23], we investigated the kinetic of p50 expression in bone marrow-derived DC (BM-DC) undergoing LPS-driven maturation. As shown in Figure 1A, a gradual increase of nuclear p50 NF-κB was observed in wt BM-DC in response to LPS treatment. Since depletion of p50 NF-κB restores production of inflammatory cytokines (e.g. TNFα) in LPS-tolerant macrophages [19], [23], we investigated whether its depletion in BM-DC could potentiate their immunostimulatory/inflammatory functions. Unstimulated or 24 hours LPS-stimulated BM-DC, loaded with the MHC II-specific OVA peptide OVA_323–339_ [29], were co-cultured for five days with CD4^+^ T cells, purified from either spleen or lymph nodes of OT-II transgenic mice, expressing the T cell receptor specific for OVA_323–339_. T cell activation was then analyzed. No relevant differences in proliferation were observed for T cell incubated with either wt or p50^−/−^ BM-DC (Figure S1C). However, T cells cultured with either untreated or LPS-treated p50^−/−^ BM-DC displayed an increased production of IFN-γ (Figure 1B). To strengthen this observation, we tested the capability of BM-DC to activate CD4^+^ T cells *in vivo*. Untreated or LPS-treated (24 hours) wt and p50^−/−^ BM-DC, loaded with the MHC II-specific OVA peptide (OVA_323–339_), were injected in the footpads of wt mice and the draining lymph nodes recovered after 7 days. As shown by ELISA, p50^−/−^ BM-DC induced higher secretion of IFN-γ in lymph nodes (Figure 1C)**,** which was also produced by CD4^+^ T cells, as confirmed by co-staining of membrane CD4 and intracellular IFN-γ (Figure 1D). In accordance, we observed increased levels of IFN-γ secretion in the supernatants obtained from spleens of mice injected with p50^−/−^ BM-DC (Figure 1E).

Next, we tested the capability of BM-DC to present antigens to CD8^+^ T cells *in vivo*. To this purpose, wt and p50^−/−^ BM-DC were loaded with the MHC I-specific OVA peptide, OVA_257–264_. Also in this case, p50^−/−^ BM-DC induced higher secretion of IFN-γ in lymph nodes and spleen (Figure 1F and H) and FACS analysis revealed that IFN-γwas secreted by CD8^+^ T cells (Figure 1G). Moreover, dexamer staining confirmed a higher number of OVA-specific T cells in the spleen of these mice (Figure 1I).

These results demonstrate that as compared to wt BM-DC, p50^−/−^ BM-DC are stronger inducers of T cell effector functions, in particular IFNγ production. To assess possible differences at the level of antigen processing, we took advantage of the DQ ovalbumin, a self-quenched conjugate of ovalbumin that becomes highly fluorescent upon proteolytic degradation [30], [31]. Wt and p50^−/−^ BM-DC were loaded with DQ ovalbumin and left in culture for a maximum of 3 hours and DQ ovalbumin fluorescence was analyzed at different time points (Figure 1L). Although p50^−/−^ BM-DC displayed a mild reduction in their antigen processing capability, no relevant differences were observed in comparison with wt BM-DC after 3 h.

### p50 Regulates the Maturation of BM-DC

**Figure 2:**
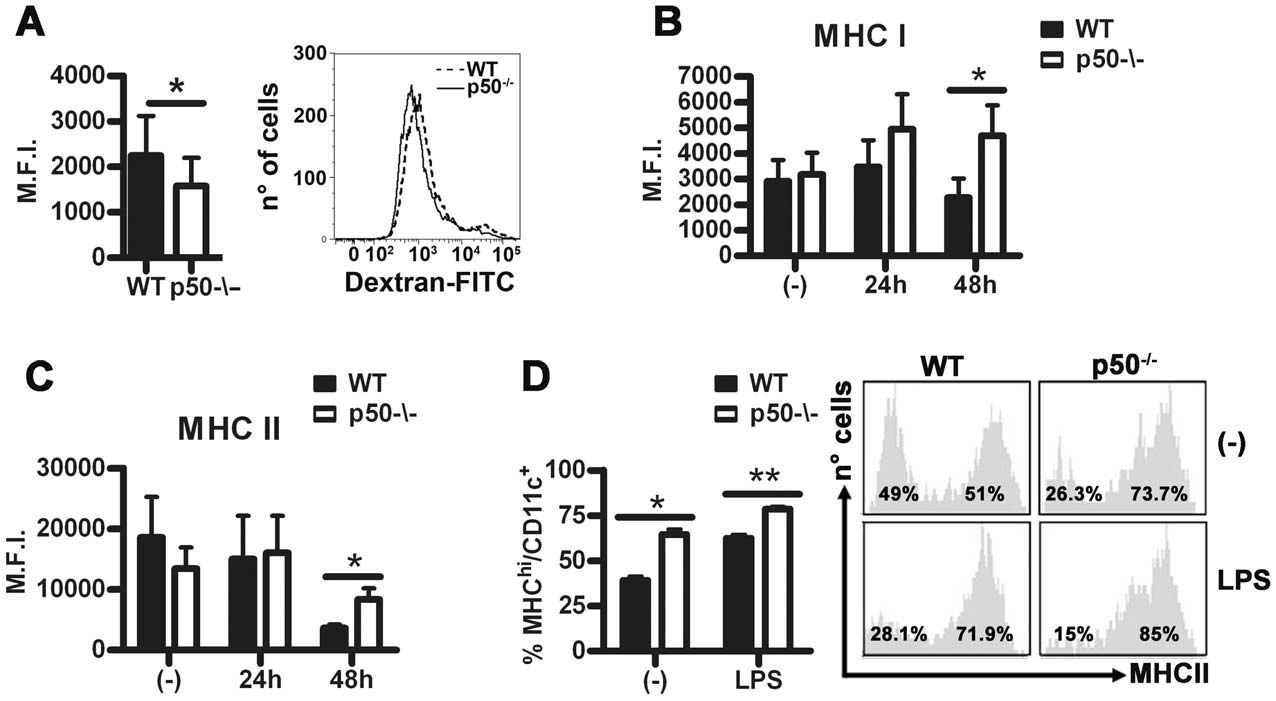
Lack of p50 increases DC maturation. (A) Endocytic activity. Left: data represent mean ± SEM of 3 independent experiments performed in triplicate. Right: representative experiment showing the shift of wt BM-DC (dashed line) compared to p50^−/−^ BM-DC (solid line) (B and C) Cytofluorimetric analysis of MHC class I and II expression by wt and p50^−/−^ BM-DC stimulated with LPS for the indicated time. Data represent mean ± SEM (N = 4). (D) Cytofluorimetric analysis of MHC II expression by wt and p50^−/−^ splenic DC, 24 h after the injection of LPS (100 ng/g mouse). Left, data from 3 experiments (6 mice/group) are shown. Right, representative panel of MHC-II high and low CD11c^+^ cells. * *P*<0.05 ** *P*<0.01, *t* test.

Since p50 accumulation inhibits the antigen-presenting capacity of BM-DC, we analyzed the effects of p50 deficiency on BM-DC maturation. We could not observe significant differences in the expression levels of both the DC marker CD11c and the LPS receptor TLR4 (Figure S1A and S1B), between wt and p50^−/−^ BM-DC**.** As high endocytic activity characterizes immature BM-DC [2], [32], we also evaluated the uptake of FITC-dextran and found a decreased endocytic activity in p50^−/−^ BM-DC (Figure 2A). A higher level of maturation of p50^−/−^ BM-DC treated with LPS for 48 hours was confirmed by the detection of increased surface levels of both MHC I and II molecules (Figure 2B and C). Further, splenic DC from p50^−/−^ mice expressed higher levels of MHC II, at the steady state as well as upon injection of mice with a sub-lethal dose of LPS (100 ng/g animal weight) (Figure 2D). In contrast, no significant changes in the expression of co-stimulatory molecules (i.e., CD80, CD86 and CD40) and CCR7 were found (Figure S1D).

### p50 Regulates Survival of LPS-matured DC

**Figure 3:**
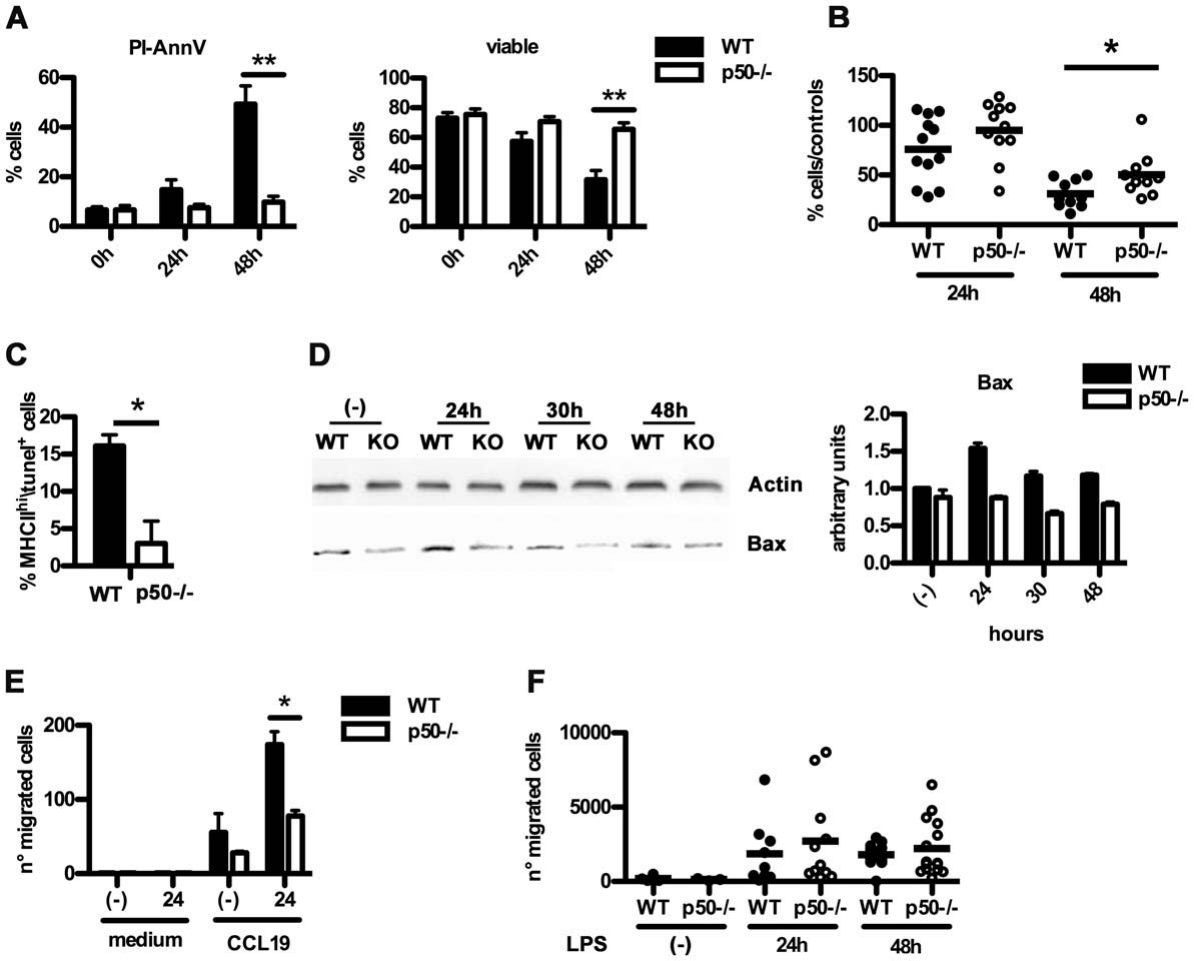
Lack of p50 increases DC survival. (A) Cytofluorimetric analysis of apoptotic BM-DC upon LPS stimulation. Data represent mean ± SEM (N = 7) (B) Cytofluorimetric analysis of splenic DC survival 24 h and 48 h after LPS injection in mice. Data represent mean ± SEM of 3 independent experiments (each dot corresponds to 1 mouse). (C) Analysis of MHC II^+^ splenic DC survival 24 h after the injection of 100 ng LPS/g (animal weight). *In situ* apoptosis of splenic DC was determined by TUNEL staining. Results are representative of 3 independent experiments (4 mice/group). (D) Bax protein levels in wt and p50^−/−^ BM-DC stimulated with LPS for the indicated time. Left panel, 1 of 2 independent experiments is shown. Right panel, mean ± SEM (N = 2). (E) *In vitro* BM-DC migration of wt and p50^−/−^ BM-DC. 24 h LPS stimulated BM-DC were seeded in a boyden chamber and migration towards CCL-19 was evaluated after 90′ incubation. (F) *In vivo* migration of wt and p50^−/−^ BM-DC to draining lymph nodes. BM-DC unstimulated or stimulated with LPS for 24 h were labeled with a fluorescent dye and injected into the footpads of wt mice. Popliteal draining lymph nodes were recovered 24 h and 48 h later and percentage of migrated BM-DC was evaluated by flow cytometry (N = 4−15). *P<0.05 ** *P*<0.01, *t* test.

DC lifespan has been previously estimated to be as short as 3 days in vivo [14], [33]. To address whether an increase in cell survival could account for the increased T cell activation by p50^−/−^ BM-DC, p50-deficient and -competent BM-DC were stimulated with LPS and then stained with a non-vital dye PI (Propidium Iodide) and an anti-AnnexinV antibody [34] (Figure 3A). After 48 hours of LPS treatment, we observed a high number of double AnnexinV/PI positive (apoptotic) wt BM-DC (60%). In contrast, LPS-treated p50^−/−^ BM-DC displayed increased survival, with only 10% of AnnexinV/PI positivity. No major differences were observed in single positive Annexin V or PI cells, leading to a strong difference in viable cells after 48 h of LPS activation (Figure 3A). To strengthen this observation, we tested the survival of wt and p50^−/−^ splenic DC exposed to LPS stimulation. As the absence of p50 NF-κB in mice confers a higher susceptibility to death in response to LPS injection [19], we first identified the dose of 100 ng/g (animal weight) as the correct amount of LPS to perform this experiment. Mice were injected with a single dose of LPS and the percentage of viable splenic DC was evaluated 24 and 48 hours later by flow cytometry (Figure 3B). As compared to wt splenic DC, splenic p50^−/−^ DC had a slower decline and a higher survival, confirming that p50 NF-κB is an important regulator of DC lifespan *in vivo*. To substantiate this observation, splenocytes from LPS-treated mice were double-stained for MHC II and TUNEL, to detect apoptosis-related DNA fragmentation. The number of MHC II^high^-TUNEL^+^ cells was higher in spleens from wt mice (Figure 3C). We then analyzed the expression of selected pro- and anti-apoptotic genes. Among the genes tested, only four were significantly different in terms of mRNA levels, between wt and p50^−/−^ BM-DC. These included the anti-apoptotic factor *PAI-2,* which was lower in wt BM-DC, and the pro-apoptotic factors *p21*, *NURR77* and *BAX*, whose levels of expression were higher in wt BM-DC (Figure S2). However, only the expression of the *BAX* gene product was increased in wt BM-DC as compared to p50^−/−^ BM-DC (Figure 3D), suggesting that p50-induced BAX expression may be relevant in the induction of BM-DC death [35].

### Lack of p50 does not Affect the DC Homing to Draining Lymph Nodes

It has been previously reported that lack of the p50 precursor p105 leads to decreased migration [36], therefore, we tested if the lack of p50 NF-κB affects BM-DC migration. First, we tested *in vitro* BM-DC migration after only 24 h activation with LPS in order to test the capability of BM-DC to migrate towards the chemokine CCL-19 when they were still viable (Figure 3E). As shown, p50^−/−^ BM-DC displayed decreased migration. To evaluate the *in vivo* significance of this observation, 24 h LPS-stimulated wt or p50^−/−^ BM-DC were labeled with a fluorescent vital dye (CMTMR) and then injected into the footpads of wt recipient mice. Next, 24 or 48 h after BM-DC injection, the number of fluorescent BM-DC reaching the draining lymph node were analyzed by flow cytometry (Figure 3F). Given that the expression of CCR7 was similar between wt and p50^−\−^ BM-DC (Figure S1D), no relevant differences were observed in lymph nodes (Figure 3F), suggesting that the increased p50^−\−^ BM-DC survival *in vivo* (Figure 3B, C) may compensate for their defective migration observed *in vitro* (Figure 3E).

### p50 Inhibits the Capacity of DC to Activate a Th1- cytokine Profile

**Figure 4:**
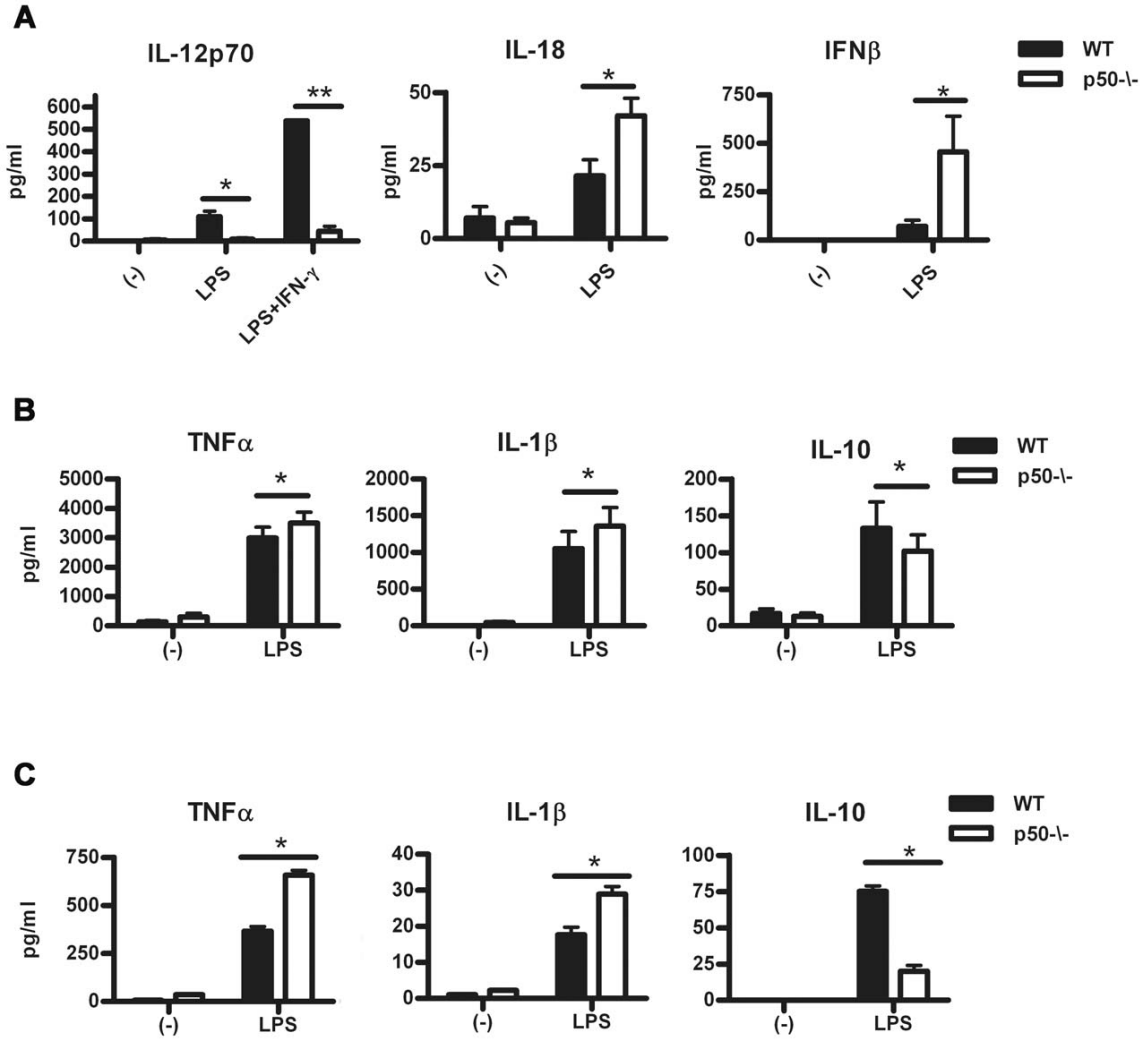
Lack of p50 in DC favors a Th1-promoting cytokine profile. (A) IL-12p70, IL-18 and IFN-β secretion by wt and p50^−/−^ BM-DC stimulated with LPS or LPS and IFN-γ for 24 h. Data represent mean ± SEM (N = 3). IL-10, TNFα and IL-1β secretion by wt and p50^−/−^ BM-derived (B) and splenic DC (C) stimulated with LPS for 24 h. Data represent mean ± SEM (N = 5 and 3, respectively). * *P*<0.05 ** *P*<0.01, *t* test.

Cytokines such as IL-12, IL-18 and type I IFNs can bias CD4^+^ T-cell priming towards a pro-inflammatory Th1 phenotype [37], [38], IL-12 being considered the key cytokine in the promotion of Th1 immunity [39]. In agreement with a previous report [40], p50^−/−^ BM-DC did not produce IL-12 upon LPS stimulation (Figure 4A). However, LPS-stimulated p50^−/−^ BM-DC expressed higher protein and mRNA levels of both IL-18 and IFN-β (Figure 4A and Figure S3A). A similar trend was observed for the expression of IFN-α (Figure S3A). To evaluate the role of IL-18 and IFN-β in promoting IFN-γ expression by T cells, we performed *in vitro* co-cultures of wt or p50^−/−^ BM-DC and T cells in the presence of either specific anti-IL-18 or anti-IFN-β blocking antibodies. Inhibition of the biological activity of either IL-18 or IFN-β partially prevented the p50^−/−^ BM-DC-mediated secretion of IFN-γ by CD4^+^ T cells, suggesting that these cytokines may compensate, at least partially, for the lack of IL-12 (Figure S3B). A previous work by Kono et al. showed that p50^−/−^ naive CD4^+^ T cells normally differentiate into Th17 cells [41]. However, p50 expression in DC has been recently suggested to prevent the activation of autoreactive T cells [42]. We investigated whether IL-23, which is involved in Th17 differentiation [43], was differentially expressed in wt vs p50^−/−^ BM-DC. As shown (Figure S3C), the production of IL-23 was strongly reduced in p50^−/−^ BM-DC. Type I IFNs mediate the innate response to viral infections and are required for a full DC response to TLR [44] and their stimulation of T and B cells [45]. In agreement with our previous observation in macrophages [19], LPS-treated p50^−/−^ BM-DC displayed higher recruitment of Polymerase II onto the *IFN-β* gene promoter and enhanced STAT-1 phosphorylation (Figure S3D and S3E). In addition, we found that p50^−/−^ BM-DC secreted higher levels of TNF-α and IL-1β, along with decreased levels of IL-10 (Figure 4B). Similar profiles were obtained for the secretion of TNF-α, IL-1β and IL-10 by splenic DC (Figure 4C).

### p50 is Required for the Immunoregulatory Functions of DC

**Figure 5:**
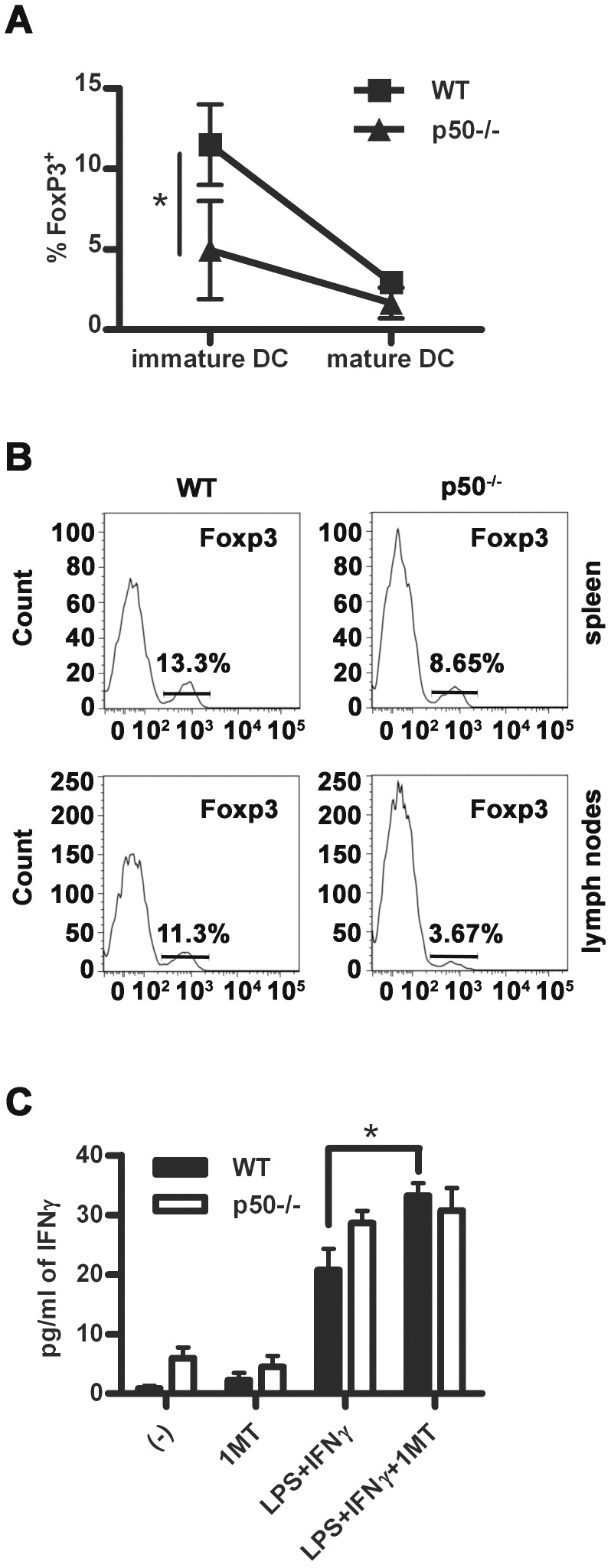
Lack of p50 impairs the immunoregulatory activity of DC. (A) *In vitro* generation of Foxp3^+^ T cells by wt and p50^−/−^ BM-DC. Data represent mean ± SEM (N = 3). (B) Cytofluorimetric analysis of naturally occurring Foxp3^+^ T cells in spleens and lymph nodes of wt and p50^−/−^ mice. Histograms show the % of Foxp3^+^ cells among CD4^+^ cells. (C) Effect of 1-methyl DL-tryptophan (1 MT; 10 µM; Sigma Aldrich) on the secretion of IFN-γ by OVA specific CD4^+^ T cell, in response to BM-DC loaded with the CD4^+^ T cell specific OVA_323–339_ peptide and treated or not for 24 h with 100 ng/ml LPS and 200 U/ml IFN-γ. Data represent 1 of 3 independent experiments. * *P*<0.05, *t* test.

Tolerogenic DC are characterized by their ability to induce differentiation of FoxP3^+^ regulatory T cells [7]. Therefore, we established an *in vitro* co-culture of BM-DC and naïve T cells and analyzed the generation of Foxp3^+^ T cells. As expected [7], immature BM-DC were more efficient in inducing CD4^+^CD25^+^ and Foxp3^+^ T cells as compared to mature BM-DC (Figure 5A). Further, wt BM-DC induced higher numbers of regulatory T cells as compared to p50^−/−^ BM-DC (Figure 5A and Figure S4A), while a higher number of CD4^+^ Foxp3^+^ cells was observed at the steady state in both spleen and lymph nodes of wt mice, as compared to p50^−/−^ mice (Figure 5B). Treg-inducing mechanisms by DC include the expression of IDO and the subsequent generation of the kynurenine metabolite of the amino acid tryptophan [46]. Noteworthy, lack of p50 resulted in reduced expression and activity of IDO (Figure S4B and S4C), the latter being measured upon splenic DC treatment with IFN-γ, the main inducer of IDO. To confirm the functional significance of this observation, we performed a BM-DC-T cell co-culture in which BM-DC were previously treated with the IDO inhibitor 1-methyl-DL-tryptophan (1-MT). As shown, levels of T cell-derived IFN-γ promoted by wt BM-DC treated with 1-MT were similar to those induced by untreated p50^−/−^ BM-DC (Figure 5C).

### Silencing of p50 Improves DC-mediated Immune Response *in vivo*

**Figure 6:**
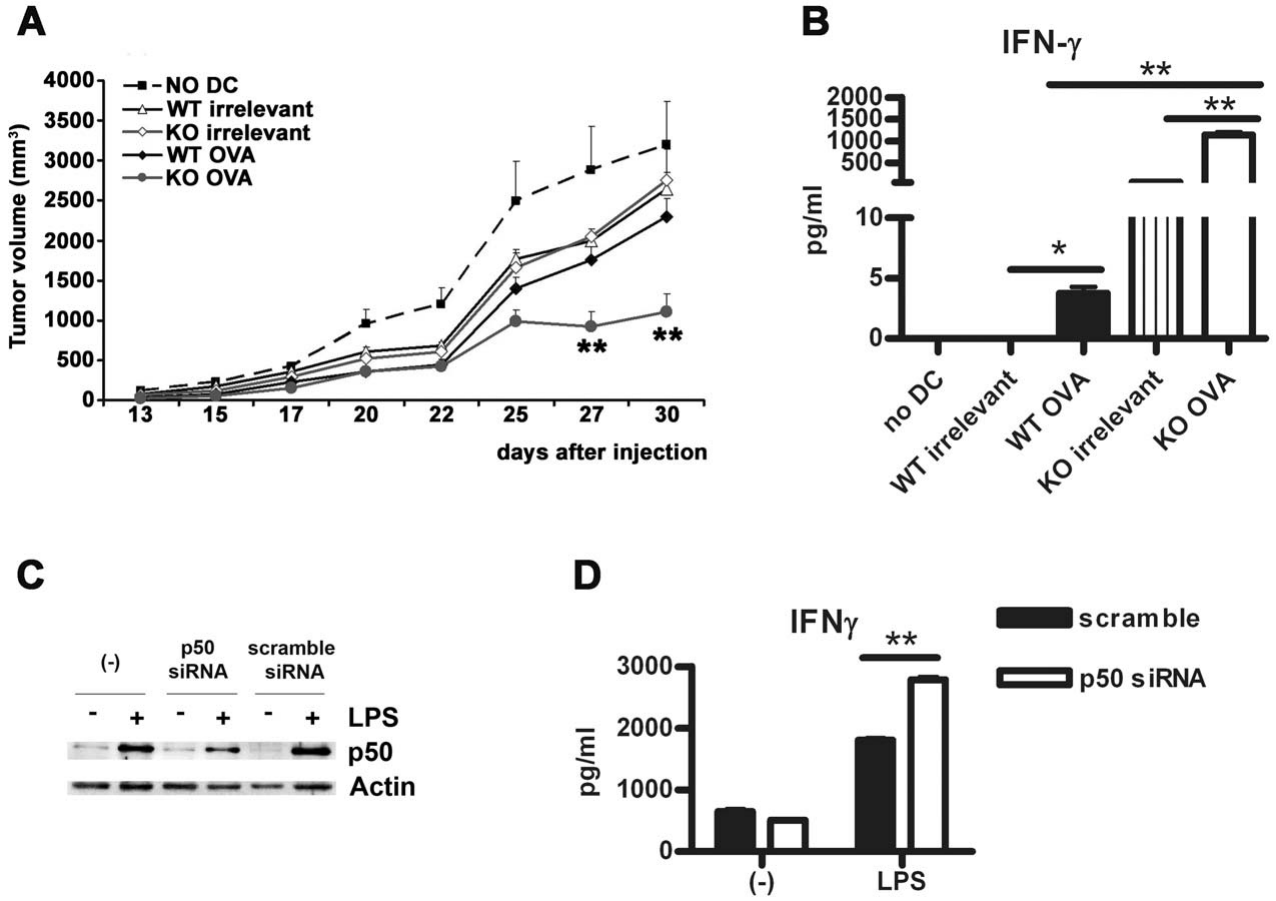
Lack of p50 improves DC vaccination ability. (A) Vaccination of wt recipient mice with wt or p50^−/−^ BM-DC loaded with either OVA or an irrelevant protein. Vaccinated mice were injected with B16-OVA melanoma cells and tumor growth evaluated at different times. Data from one of two independent experiments (6 mice/group) are shown. (B) *Ex vivo* IFN-γ secretion by splenocytes recovered at day 30 after vaccination. Data represent the mean ± SEM of 6 mice/group. (C, D) Human mo-DC were transfected with a p50-specific Stealth RNAi siRNA (p50 siRNA) or with a Stealth negative control (scramble), incubated with or without LPS (100 ng/ml) for 48 h. Next cells were washed and lysed to analyzed p50 levels by western blotting (C) or added to purified allogeneic T cells (ratio mo-DC:T cells 1∶10) (D). After 5 days, supernatants were collected and tested by ELISA. Data represent mean ± standard deviation of 1 of 3 independent experiments performed in triplicate. * *P*<0.05, ** *P*<0.01, *t* test.

Altogether, our data suggest that accumulation of p50 NF-κB in mature DC may promote their tolerogenic functions by shortening their lifespan, enhancing expression of IDO, enhancing their capacity to promote expansion of Foxp3^+^ Treg cells and by limiting their capacity to induce Th1 immunity dominated by IFN-γ [37], [38]. Based on this, we tested the effects of vaccination with p50^−/−^ BM-DC in an *in vivo* model of melanoma, using ovalbumin antigen-expressing B16 melanoma cells [47]. To this aim, we incubated BM-DC, obtained from either wt or p50 KO mice, with ovalbumin to allow the internalization and the processing of the protein. As control, BM-DC were incubated with BSA. After 6 h, BM-DC were activated with LPS for additional 24 hours and then injected *in vivo*. BM-DC injection was followed by injection of OVA-expressing B16 melanoma cells. As shown in Figure 6A, vaccination with p50^−/−^ BM-DC produced a stronger inhibition of melanoma growth, along with increased production of IFN-γ in the spleen (Figure 6B). Of note, as compared to wt BM-DC, p50^−/−^ BM-DC induced high levels of IFN-γ even when loaded with the irrelevant antigen. This effect could be attributed to the higher levels of inflammatory cytokines (Figure 4), MHC I and MHC II molecules (Figure 2) and decreased levels of IL-10 expressed by p50^−/−^ BM-DC, which would better support T cell activation. Finally, to evaluate whether silencing of p50 could improve the capacity of human DC in activating T cell functions, human monocyte-derived DC (mo-DC) were transfected with a RNAi siRNA duplex specific for p50 and then tested in a mixed leukocyte reaction. Similarly to what observed for p50 KO mouse BM-DC, p50-silenced mo-DC (Figure 6C) induced higher IFNγ production by co-cultured allogeneic lymphocytes (Figure 6D). These results demonstrate that inhibition of p50 NF-κB in DC improves vaccination efficacy and support a potential adjuvant role for p50 manipulation in DC-based vaccination protocols.

## Discussion

The observation that nuclear p50 NF-κB controls DC survival, tolerogenic as well as immunogenic functions indicates that p50 is a major determinant of both innate and adaptive immune responses and underpins its relevance in diseases characterized by aberrant immune responses, including infection, transplantation and cancer. Tolerance is a key mechanism preventing harmful immune effector reactions [12], [19] and DC play central roles in both central (thymic) and peripheral tolerance [7]. Current data suggest that DC maturation provides the critical switch to deliver the signals for inducing effector T cell development and memory, rescuing T cells from apoptosis to protective immunity [1], [2], [7]. However, in the absence of inflammation, an incomplete form of DC maturation generates a tolerogenic antigen-presenting cell, such that lymph node and spleen DC induce tolerance in naïve T cells [48]. Here we show that lack of p50 in DC enhances their lifespan, maturation and capacity of activating and expanding CD4^+^ and CD8^+^ T cells *in vivo*, suggesting that the nuclear accumulation of the p50 NF-κB subunit, as occurring during LPS-driven DC maturation, provides a feedback mechanism to limit the DC capacity to perpetuate the immune response. This notion is strengthen by the observations that p50 accumulation in DC is paralleled by enhanced expression of tolerogenic molecules (eg. IDO) and decreased levels of pro-inflammatory cytokines (IL-1, IL-18, IFN-β), which concur to promote tolerance. The association between p50 and tolerogenic functions of DC is further underlined by the observation that p50 NF-κB deficient DC are poor inducer of Foxp3^+^ suppressor T cells differentiation [49]. The inhibitory action of p50 is shared by other components of the immune system [19], [22], [50], [51]. As an example, accumulation of the p50 NF-κB homodimer in monocyte/macrophages was described to mediate their tolerance to LPS and to play protective role in sepsis [23]. Similarly, nuclear accumulation of p50 NF-κB in tumor-associated macrophages (TAM) was associated with both defective M1 inflammation and development of M2 macrophage polarization, associated with a suppressive protumoral phenotype [19], [22]. Moreover, while in lymphoid cells p50 was reported to be essential for the expression of Th2 cytokines (eg. IL-4) [50], expression of p50 NF-κB by DC was reported to promote optimal Th2 cell differentiation [51] **.** It is tempting to speculate that nuclear accumulation of p50 NF-κB in different immune cell types provides a general mechanism to brake both the inflammatory and immune responses, triggered by infections and inflammatory signals (e.g. LPS), which would be instrumental to both the resolution phase of inflammation and restoration of tissue homeostasis. In this perspective, it is worth to know that p50 nuclear accumulation is promoted also by anti-inflammatory mediators, such as IL-10, TGFβ and PGE2 [22] which are part of the resolution phase of inflammation [52].

Noteworthy, inhibition of p50 promotes DC maturation and their capacity of activating and expanding CD8^+^ T cells *in vivo*, a main goal of immunotherapy [53]. In this regard, the NLRP3 inflammasome complex is one of the molecular targets of adjuvants and it is required for adjuvanticity in dendritic cell-based vaccines [54], [55], where adjuvant-mediated activation of both TLR and the inflammasome pathway [55], [56] promotes IL-18 and IL-1β-dependent antitumor immunity [54], [57]. Interestingly, we observed that p50 NF-κB acts as a negative regulator of both the caspase 1 (unpublished data) activity and the subsequent release of IL-1β.

The observation that lack of p50 increases DC lifespan may be particularly relevant for clinical applications of current DC-based vaccines, as the short lifespan of DC, as well as their incomplete maturation, represent serious limitations [58], [59]. As proof of concept, we demonstrated that vaccination of melanoma-bearing mice with antigen-pulsed LPS-treated p50^−/−^ DC boosted antitumor immunity and inhibition of tumor growth, while silencing of p50 in human DC enhanced their ability to induce a more powerful Th1 activation.

Our data support and integrate recent observations proposing that expression of p50 NF-κB in immature DC is essential to prevent activation of autoreactive T cells [42]. We demonstrate that p50 NF-κB orchestrates DC functions and survival in response to an inflammatory signal (LPS) during their maturation process, thus providing a homeostatic mechanism tuning the balance between uncontrolled activation of adaptive immunity and immune tolerance.

## Supporting Information

Figure S1 **Effect of p50 NF-κB on both differentiation and LPS-driven maturation of BM-DC.** (A) Cytofluorimetric analysis of CD11c expression during the differentiation of wt and p50^−/−^ DC from whole bone marrow cells. Cells were seeded (p1), harvested and analyzed by flow cytometry every 3 days up to 9 days (exp). (B) Real-time PCR analysis of TLR4 mRNA expression by wt and p50^−/−^ BM-DC. Data represent mean ± SEM (N = 3). (C) Analysis of T cell proliferation induced by wt and p50^−/−^ DC. CD4^+^ T cells were purified from the spleen of OT-II mice and co-cultured with wt or p50^−/−^ LPS-treated DC loaded with a class II-restricted peptide. Each group was performed in triplicate. [^3^H]Thymidine incorporation was measured on day 5 after a 16-h pulse. A representative experiment of 2 independent experiments with similar results is shown. (D) Cytofluorimetric analysis of maturation markers CD80, CD86, CD40 and CCR7 expression by wt and p50^−/−^ BM-DC stimulated with LPS for the indicated time. Data represent mean ± SEM (N = 10). anti-CD86 (clone GL1) and anti-CCR7 (clone 4B12) were from e-Bioscience, San Diego, CA; anti-CD80 (clone 16-10A1), anti-CD40 (clone 3/23), and CCR7 (clone 4B12) were from BD Biosciences, San Diego, CA.
(TIF)

Figure S2 **Regulation of pro- and anti-apopototic genes by wt and p50^−/−^ BM-DC.** Real-time PCR analysis of BAX, p21, NURR77, and PAI2 mRNA expression in wt and p50^−/−^ BM-DC stimulated with LPS for the indicated time. Primer sequences are available upon request. Graphs represent the means of 3 independent experiments. * *P*<0.05, *t* test.
(TIF)

Figure S3 **Lack of p50 NF-κB in DC promotes enhanced Th1 differentiation via increased type I IFN and IL-18 production.** (A) Real-time PCR analysis of IL-12p40, IL-12p35, IL-18, IFN-β and IFN-α mRNA expression by wt and p50^−/−^ BM-DC stimulated for 24 hours with 100 ng/ml LPS alone or in combination with 200 U/ml IFN-γ. Data represent mean ± SEM (N = 3). (B) Effect of anti-IL-18 and anti-IFN-β antibody on the secretion of IFN-γ by OVA-specific CD4^+^ T cell, in response to BM-DC loaded with the CD4^+^ T cell specific OVA_323–339_ peptide and activated 24 hours with 100 ng/ml LPS (iso =  isotype control antibody). Data represent mean ± SEM (N = 3). Neutralizing rabbit polyclonal antibody against mouse IFN-β (5µg/ml) was from PBL Biomedical Laboratories; neutralizing rabbit polyclonal antibody against mouse IL-18 (5 µg/ml) was from MBL (Woburn, MA). (C). IL-23 secretion by wt and p50^−/−^ BM-DC. BM-DC were stimulated with 100 ng/ml LPS for 24 h, supernatants were collected and tested by ELISA. Data represent mean ± SEM (N = 3). (D) Negative regulation of IFN-β gene transcription by p50 NF-κB. Wt and p50^−/−^ BM-DC were stimulated with LPS for the indicated time. Recruitment of Polymerase II by the IFN-β promoter was analyzed by chromatin immunoprecipitation (ChIP). A total of 30×10^6^ cells were used for ChIP analysis, as previously described [19]. Primer sequences are available upon request. (E) STAT1 phosphorylation in wt and p50^−/−^ BM-DC. Wt and p50^−/−^ BM-DC were stimulated with LPS for 90 min or with IFN-γ for 15 min and total extracts analyzed with specific anti-phospho STAT1 antibody (Cell Signaling Technology, Danvers, MA). Left, one of 3 independent experiments with similar results is shown. Right, mean ± SEM (N = 3).
(TIF)

Figure S4 **Impaired IDO expression and differentiation of FoxP3+ regulatory T cells by p50^−/−^ DC.** (A) *In vitro* generation of Foxp3^+^ cells. Wt and p50^−/−^ BM-DC were co-cultured with T cells (1∶3 ratio) for 5 days. Percentages of Foxp3^+^ cells were evaluated by membrane and intracellular staining with anti-CD4, anti-CD25 and anti-Foxp3 specific antibodies. Results are representative of 3 independent experiments. (B) Real-time PCR analysis of IDO expression by wt and p50^−/−^ BM-DC. BM-DC were stimulated with either 100 ng/ml LPS or 200 U/ml of IFN-γ for the indicated time. One of 3 independent experiments with similar results is shown. (C) Kynurenine production by wt and p50^−/−^ DC. Splenic DC were seeded at the concentration of 10^6^/ml and stimulated with IFN-γ for 72 hours. Supernatants were collected and tested for the presence of the kynurenine metabolite by high-performance liquid chromatography (HPLC) as previously described [60]. * *P*<0.05, *t* test.
(TIF)
