# Supplementary figures and images for: The p50 Subunit of NF-κB Orchestrates Dendritic Cell Lifespan and Activation of Adaptive Immunity
Source: PLoS One. 2012 Sep 25;7(9):e45279. doi: 10.1371/journal.pone.0045279 (PMC3458114; doi:10.1371/journal.pone.0045279)

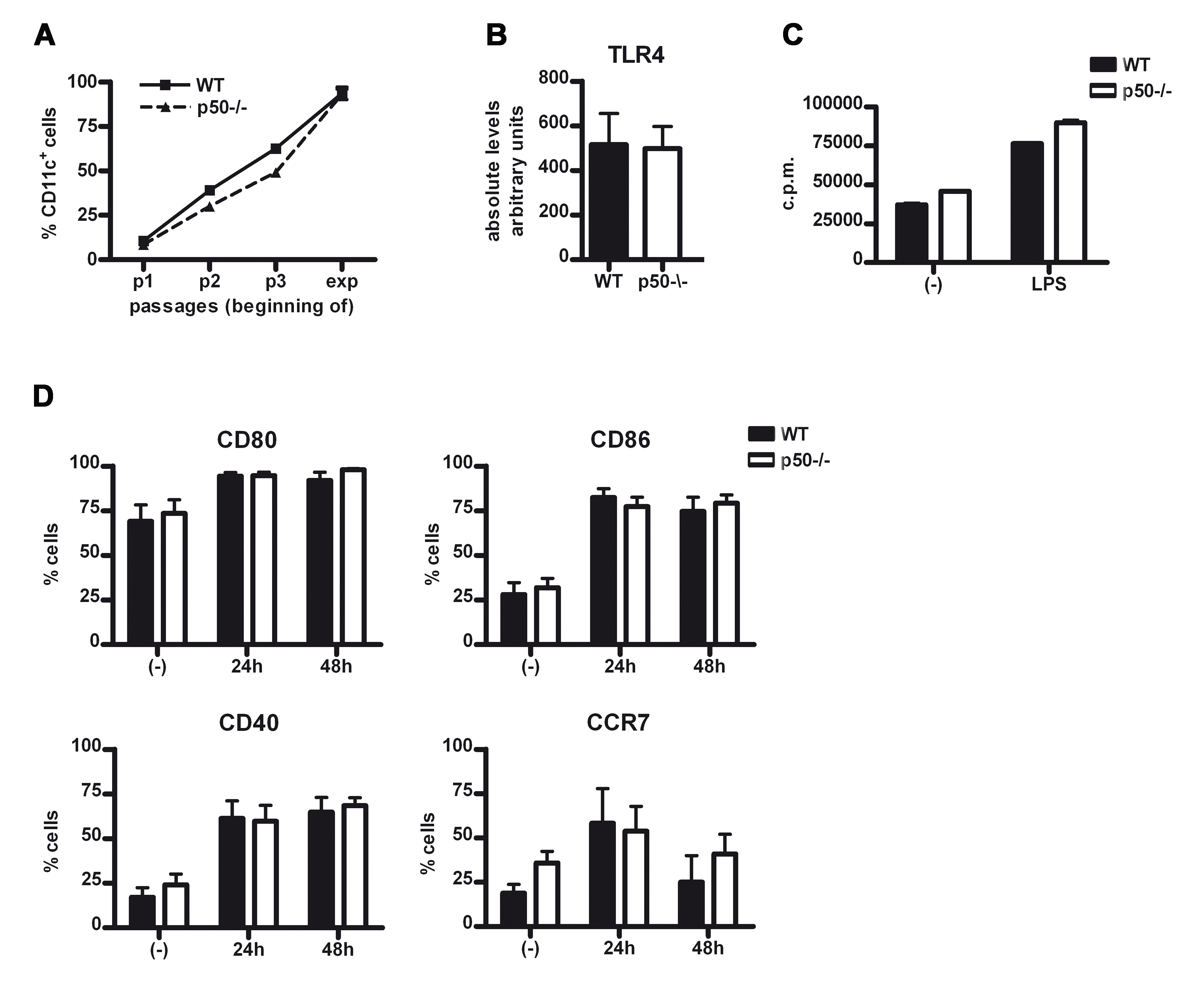

Supplement: Figure S1 — Effect of p50 NF-κB on both differentiation and LPS-driven maturation of BM-DC. (A) Cytofluorimetric analysis of CD11c expression during the differentiation of wt and p50−/− DC from whole bone marrow cells. Cells were seeded (p1), harvested and analyzed by flow cytometry every 3 days up to 9 days (exp). (B) Real-time PCR analysis of TLR4 mRNA expression by wt and p50−/− BM-DC. Data represent mean ± SEM (N = 3). (C) Analysis of T cell proliferation induced by wt and p50−/− DC. CD4+ T cells were purified from the spleen of OT-II mice and co-cultured with wt or p50−/− LPS-treated DC loaded with a class II-restricted peptide. Each group was performed in triplicate. [3H]Thymidine incorporation was measured on day 5 after a 16-h pulse. A representative experiment of 2 independent experiments with similar results is shown. (D) Cytofluorimetric analysis of maturation markers CD80, CD86, CD40 and CCR7 expression by wt and p50−/− BM-DC stimulated with LPS for the indicated time. Data represent mean ± SEM (N = 10). anti-CD86 (clone GL1) and anti-CCR7 (clone 4B12) were from e-Bioscience, San Diego, CA; anti-CD80 (clone 16-10A1), anti-CD40 (clone 3/23), and CCR7 (clone 4B12) were from BD Biosciences, San Diego, CA. (TIF) [file pone.0045279.s001.tif]

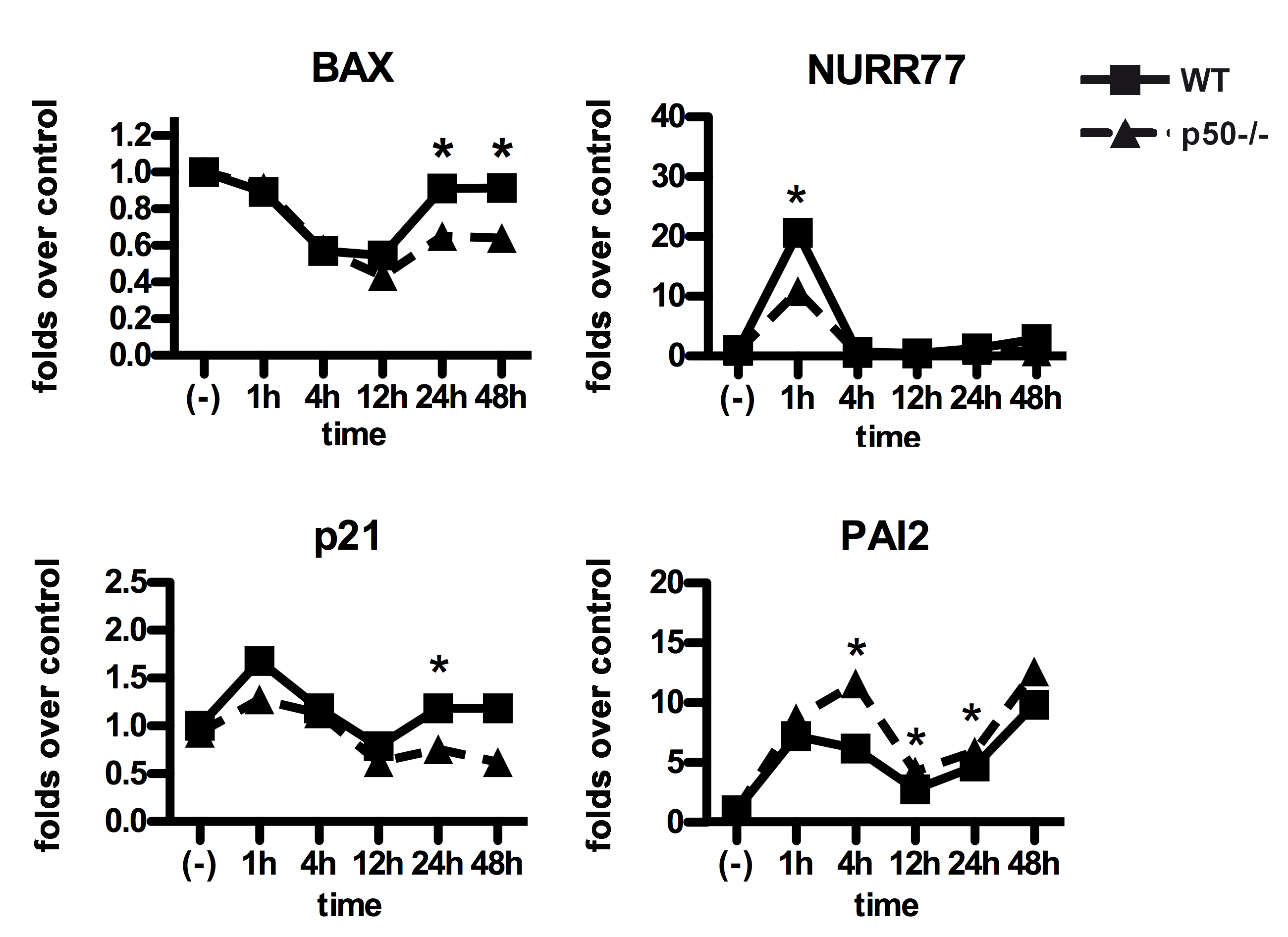

Supplement: Figure S2 — Regulation of pro- and anti-apopototic genes by wt and p50−/− BM-DC. Real-time PCR analysis of BAX, p21, NURR77, and PAI2 mRNA expression in wt and p50−/− BM-DC stimulated with LPS for the indicated time. Primer sequences are available upon request. Graphs represent the means of 3 independent experiments. * P<0.05, t test. (TIF) [file pone.0045279.s002.tif]

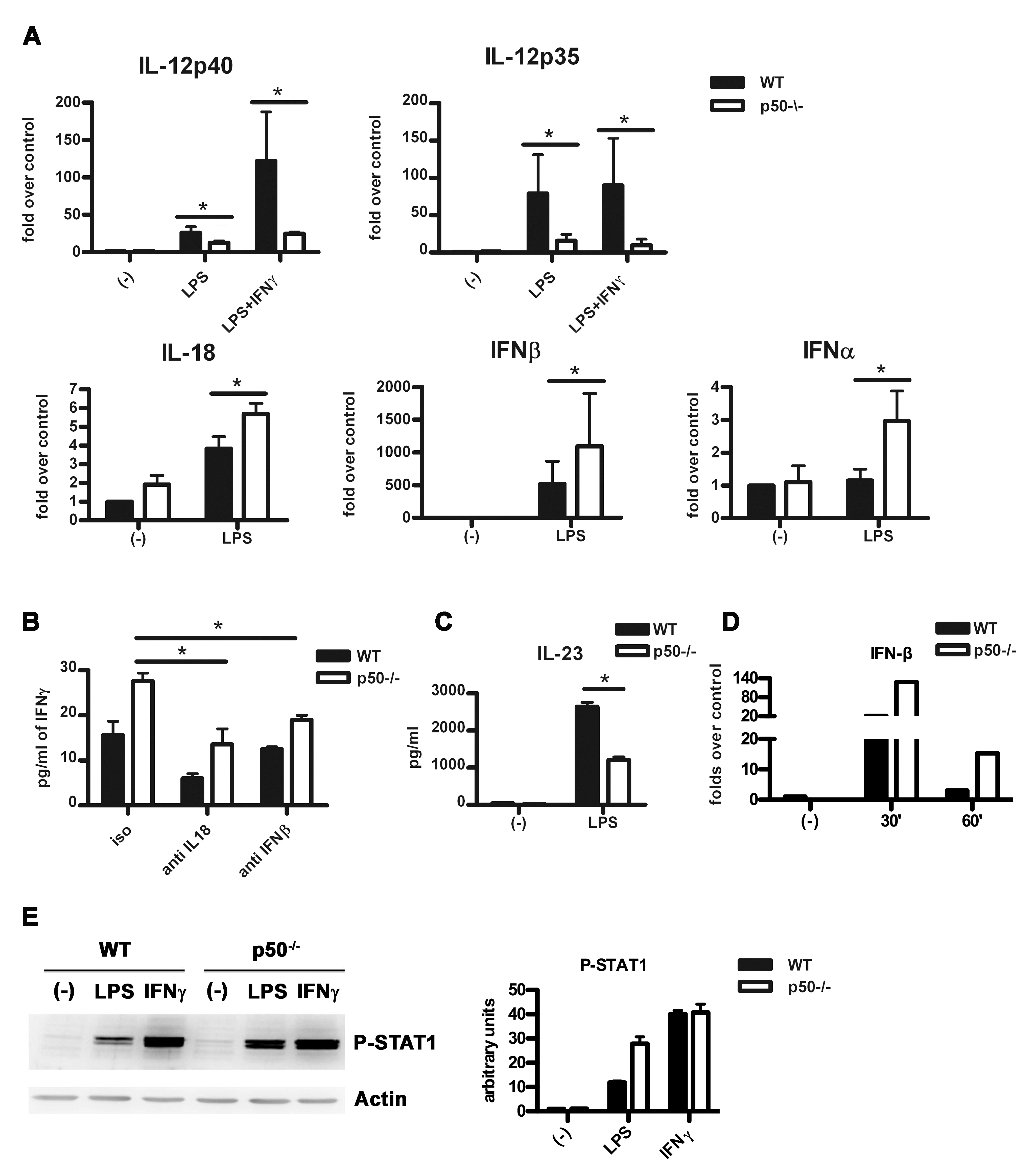

Supplement: Figure S3 — Lack of p50 NF-κB in DC promotes enhanced Th1 differentiation via increased type I IFN and IL-18 production. (A) Real-time PCR analysis of IL-12p40, IL-12p35, IL-18, IFN-β and IFN-α mRNA expression by wt and p50−/− BM-DC stimulated for 24 hours with 100 ng/ml LPS alone or in combination with 200 U/ml IFN-γ. Data represent mean ± SEM (N = 3). (B) Effect of anti-IL-18 and anti-IFN-β antibody on the secretion of IFN-γ by OVA-specific CD4+ T cell, in response to BM-DC loaded with the CD4+ T cell specific OVA323–339 peptide and activated 24 hours with 100 ng/ml LPS (iso = isotype control antibody). Data represent mean ± SEM (N = 3). Neutralizing rabbit polyclonal antibody against mouse IFN-β (5µg/ml) was from PBL Biomedical Laboratories; neutralizing rabbit polyclonal antibody against mouse IL-18 (5 µg/ml) was from MBL (Woburn, MA). (C). IL-23 secretion by wt and p50−/− BM-DC. BM-DC were stimulated with 100 ng/ml LPS for 24 h, supernatants were collected and tested by ELISA. Data represent mean ± SEM (N = 3). (D) Negative regulation of IFN-β gene transcription by p50 NF-κB. Wt and p50−/− BM-DC were stimulated with LPS for the indicated time. Recruitment of Polymerase II by the IFN-β promoter was analyzed by chromatin immunoprecipitation (ChIP). A total of 30×106 cells were used for ChIP analysis, as previously described [19]. Primer sequences are available upon request. (E) STAT1 phosphorylation in wt and p50−/− BM-DC. Wt and p50−/− BM-DC were stimulated with LPS for 90 min or with IFN-γ for 15 min and total extracts analyzed with specific anti-phospho STAT1 antibody (Cell Signaling Technology, Danvers, MA). Left, one of 3 independent experiments with similar results is shown. Right, mean ± SEM (N = 3). (TIF) [file pone.0045279.s003.tif]

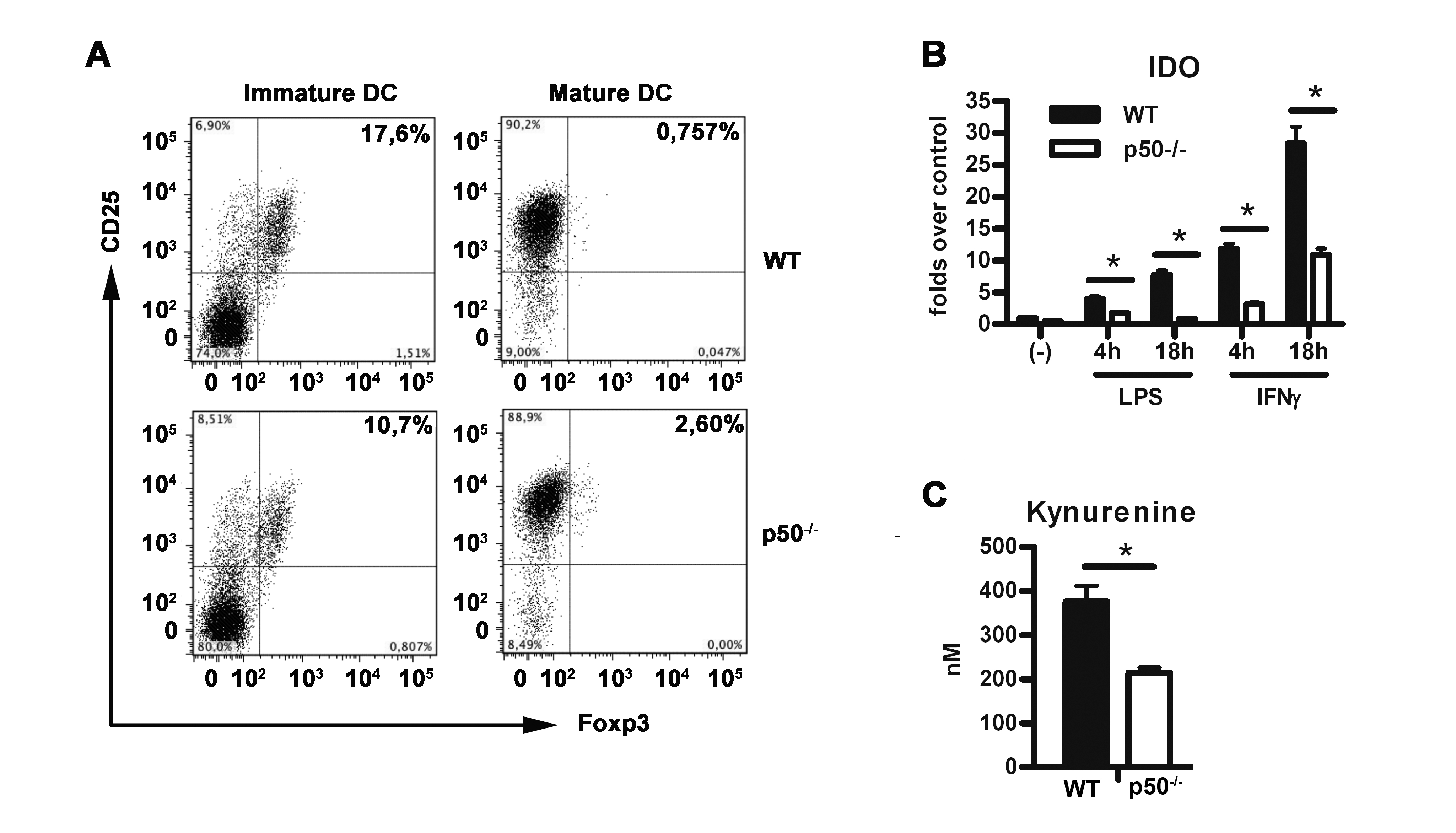

Supplement: Figure S4 — Impaired IDO expression and differentiation of FoxP3+ regulatory T cells by p50−/− DC. (A) In vitro generation of Foxp3+ cells. Wt and p50−/− BM-DC were co-cultured with T cells (1∶3 ratio) for 5 days. Percentages of Foxp3+ cells were evaluated by membrane and intracellular staining with anti-CD4, anti-CD25 and anti-Foxp3 specific antibodies. Results are representative of 3 independent experiments. (B) Real-time PCR analysis of IDO expression by wt and p50−/− BM-DC. BM-DC were stimulated with either 100 ng/ml LPS or 200 U/ml of IFN-γ for the indicated time. One of 3 independent experiments with similar results is shown. (C) Kynurenine production by wt and p50−/− DC. Splenic DC were seeded at the concentration of 106/ml and stimulated with IFN-γ for 72 hours. Supernatants were collected and tested for the presence of the kynurenine metabolite by high-performance liquid chromatography (HPLC) as previously described [60]. * P<0.05, t test. (TIF) [file pone.0045279.s004.tif]
